# Synthesis, mol­ecular and crystal structure of [(NH_2_)_2_CSSC(NH_2_)_2_]_2_[RuBr_6_]Br_2_·3H_2_O

**DOI:** 10.1107/S2056989024006832

**Published:** 2024-07-23

**Authors:** Olga V. Rudnitskaya, Milena R. Komarovskikh, Maria G. Pekarskaya, Daniil S. Pshenichnyy, Mehmet Akkurt, Ali N. Khalilov, Ajaya Bhattarai, Ibrahim G. Mamedov

**Affiliations:** a RUDN University, 115419 Moscow, Russian Federation; b Department of Physics, Faculty of Sciences, Erciyes University, 38039 Kayseri, Türkiye; c "Composite Materials" Scientific Research Center, Azerbaijan State Economic University (UNEC), Murtuza Mukhtarov str. 194, Az 1065, Baku, Azerbaijan; d Department of Chemistry, Baku State University, Z. Khalilov str. 23, Az, 1148, Baku, Azerbaijan; e Department of Chemistry, M.M.A.M.C (Tribhuvan University) Biratnagar, Nepal; Universidad de la Repüblica, Uruguay

**Keywords:** crystal structure, ruthenium halido complexes, anions, cations, αα′-(di­thio­bis­formamidinium) cation

## Abstract

The title compound, bis­[di­thio­bis­(formamidinium)] hexa­bromido­ruthenium dibromide trihydrate, [(NH_2_)_2_CSSC(NH_2_)_2_]_2_[RuBr_6_]Br_2_·3H_2_O, crystallizes in the ortho­rhom­bic system, space group *Cmcm*, *Z* = 4. The [RuBr_6_]^2−^ anionic complex has an octa­hedral structure. The Ru—Br distances fall in the range 2.4779 (4)–2.4890 (4) Å. The S—S and C—S distances are 2.0282 (12) and 1.783 (2) Å, respectively. The H_2_O mol­ecules, Br^−^ ions, and NH_2_ groups of the cation are linked by hydrogen bonds. The conformation of the cation is consolidated by intra­molecular O—H⋯Br, O—H⋯O, N—H⋯Br and N—H⋯O hydrogen bonds. The [(NH_2_)_2_CSSC(NH_2_)_2_]^2+^ cations form a hydrogen-bonded system involving the Br ^−^ ions and the water mol­ecules. Two Br ^−^ anions form four hydrogen bonds, each with the NH_2_ groups of two cations, thus linking the cations into a ring. The rings are connected by water mol­ecules, forming N—H⋯O—H⋯Br hydrogen bonds.

## Abstract 2

The [RuBr_6_]^2−^ anionic complex has an octa­hedral structure. The Ru—Br distances fall in the range 2.4779 (4)–2.4890 (4) Å. The S—S and C—S distances are 2.0282 (12) and 1.783 (2) Å, respectively. The H_2_O mol­ecules, Br^−^ ions, and NH_2_ groups of the cation are linked by hydrogen bonds.

## 1. Chemical context

Oxidation of thio­carbamide in an acidic medium results in the αα′-­(di­thio­bis­formamidinium) cation {[(NH_2_)_2_CSSC(NH_2_)_2_]^2+^ or [S_2_C_2_(NH_2_)_4_]^2+^ in a simplified form; Preisler & Berger, 1947 ▸}. There are only a few examples of compounds containing [S_2_C_2_(NH_2_)_4_]^2+^ cations described in the literature. For example, direct inter­action of compounds with [S_2_C_2_(NH_2_)_4_]Cl_2_ in concentrated hydro­chloric acid produced [S_2_C_2_(NH_2_)_4_][*M*Cl_4_], *M* = Cu, Co, Zn, Hg (Golovnev *et al.*, 2013 ▸), [S_2_C_2_(NH_2_)_4_]_2_[Hg_2_Cl_8_] (Vasiliev *et al.*, 2013 ▸) and [S_2_C_2_(NH_2_)_4_]_2_[Os^IV^Cl_6_]Cl_2_·3H_2_O (Rudnitskaya *et al.*, 2019 ▸), while changing the reaction medium to concentrated hydro­bromic acid resulted in the formation of [S_2_C_2_(NH_2_)_4_][HgBr_4_] (Golovnev *et al.*, 2013 ▸).

From the point of view of synthetic coordination chemistry, the reactions of rhenium and osmium complexes with thio­carbamide are of inter­est. These reactions led to compounds with an outer sphere di­thio­bis­formamidinium cation. Thus, the inter­action of ReO_4_^−^ with thio­carbamide (tu) in hydro­chloric acid allows the preparation of complexes [S_2_C_2_(NH_2_)_4_][ReCl_4_(H_2_O)O], [S_2_C_2_(NH_2_)_4_]_2_[ReCl_5_(H_2_O)]Cl_3_·2H_2_O and [S_2_C_2_(NH_2_)_4_]_2_[ReCl_5_(tu)]Cl (Lis, 1979 ▸, 1980 ▸; Lis & Starynowicz, 1985 ▸). In this case, oxidation of thio­urea (hereinafter, ***tu***) occurs by rhenium(VII). When K_2_[ReCl_6_] reacts with ***tu*** in dilute HCl, thio­carbamide oxidation occurs under the influence of atmospheric oxygen, giving [S_2_C_2_(NH_2_)_4_]_2_[ReCl_6_]Cl_2_·3H_2_O (Lis & Starynowicz, 1985 ▸). Similar complexes [S_2_C_2_(NH_2_)_4_]_2_[Os*X*_6_]*X*_2_·3H_2_O where *X* = Cl, Br were obtained by the reaction of H_2_[Os*X*_6_] and ***tu*** in concentrated HCl and HBr, respectively. In the aforementioned case, thio­carbamide was oxidized by osmium(IV) (Rudnitskaya *et al.*, 2008 ▸, 2019 ▸). The mol­ecular and crystal structures of the rhenium and osmium complexes discussed above were established by X-ray diffraction. The inter­action of K_4_[Ru_2_OCl_10_] with α,α′-­(di­thio­bis­formamidinium) chloride forms the unique ruthenium(III) compound [Ru_2_(tu)_3_Cl_6_]·2H_2_O, containing three ***tu*** bridging mol­ecules (Rudnitskaya *et al.*, 2017*a* ▸). The structure of [Cl_3_Ru(tu)_3_RuCl_3_] will be published elsewhere.
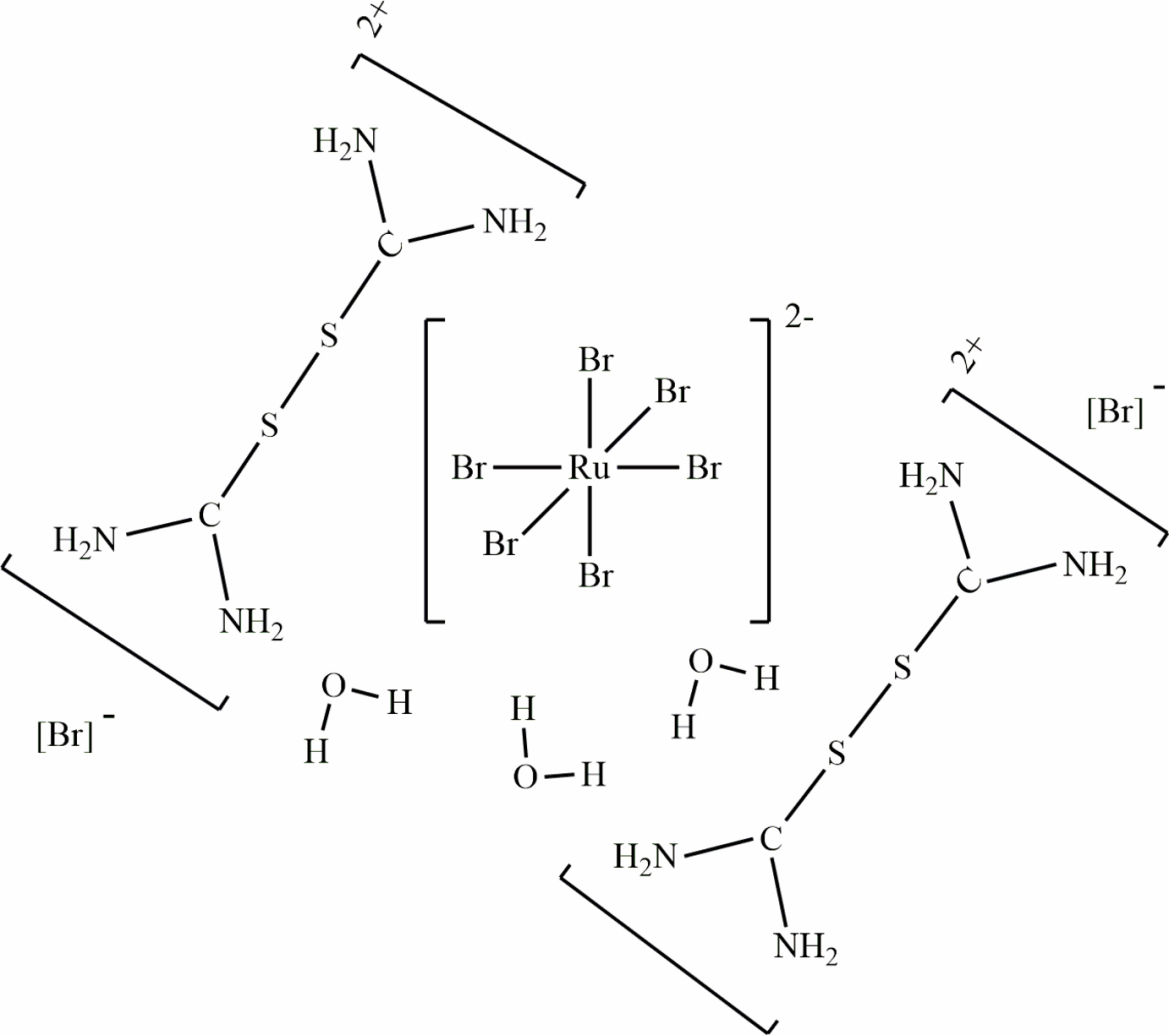


This study aimed to investigate the inter­action between ruthenium compounds and αα′-bis­(di­thio­bis­formamidinium) bromide in hydro­bromic acid solutions.

## 2. Structural commentary

**Figure 1:**
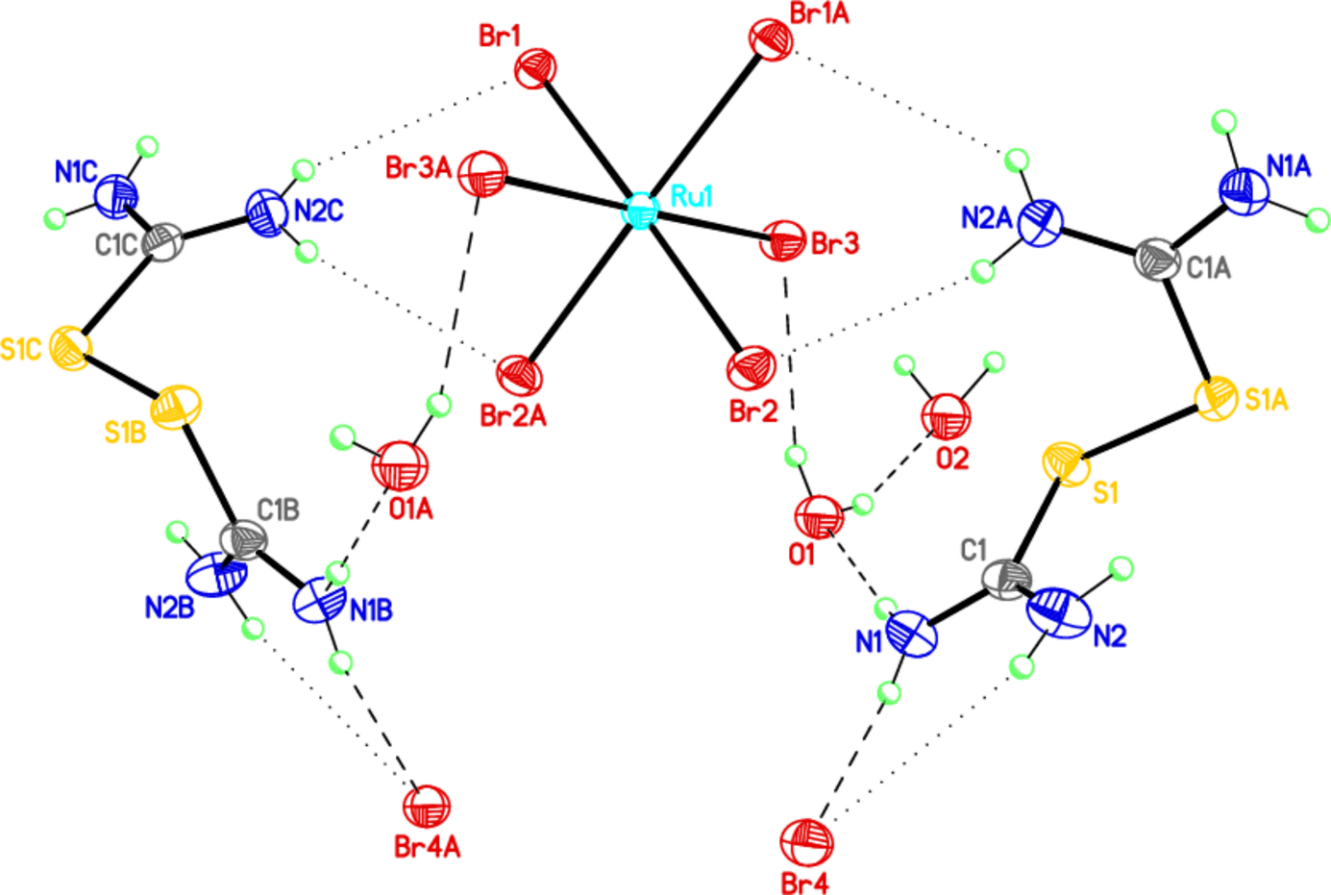
The mol­ecular structure of [S_2_C_2_(NH_2_)_4_]_2_[RuBr_6_]Br_2_·3H_2_O, showing the atom labeling and displacement ellipsoids drawn at the 30% probability level. Symmetry codes: (A) *x*, *y*, 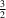 − *z*; (B) 1 − *x*, *y*, 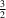 − *z*; (C) *x*, 1 − *y*, 1 − *z*.

The title compound (Fig. 1 ▸) is isostructural to the similar osmium complex (Rudnitskaya *et al.*, 2008 ▸, 2019 ▸). In the α,α′-­(di­thio­bis­formamidinium) cation, the S—S bond is single and has a length of 2.0282 (12) Å, while in the osmium complex the length of this bond is 2.039 (2) Å. The cation adopts the most energetically favorable gauche conformation with a C—S—S—C torsion angle of 97.15 (11)°, which is slightly smaller than the angle in the osmium analogue (102.62°). The intrinsic symmetry of the cations is *C*2, and the thio­carbamide fragments retain a planar structure in both complexes. The intrinsic symmetry of the [RuBr_6_]^2–^ ion is *D*2*h*. The coordination number of ruthenium is 6, and the anion takes the form of a distorted octa­hedron, *d*(Ru—Br) = 2.4779 (4)–2.4890 (4) Å for three Br atoms are different, but within standard errors (Table 1 ▸). The conformation of the cation is aslo consolidated by intra­molecular N—H⋯S hydrogen bonds (Table 2 ▸, Fig. 1 ▸). In [(NH_2_)_2_CSSC(NH_2_)_2_]_2_[OsBr_6_]Br_2_·3H_2_O, the conformation of the cation is also stabilized by intra­molecular N—H⋯S hydrogen bonds (Rudnitskaya *et al.*, 2008 ▸). The geometric parameters of the title compound are normal and consistent with those of the related compounds described in the *Database survey* (Section 4).

## 3. Supra­molecular features

The [(NH_2_)_2_CSSC(NH_2_)_2_]^2+^ cations and [RuBr_6_]^2–^ complex anion form a system of hydrogen bonds with the Br^−^ ions and water mol­ecules (Table 2 ▸, Figs. 1 ▸ and 2 ▸). Two cations are bound in a ring by two Br^−^ ions, each forming four hydrogen bonds with the NH_2_ groups of the cations. A similar system of hydrogen bonds is present in the osmium complex.

For two O—H⋯Br hydrogen bonds [O1—H1*C*⋯Br3 and O2—H2⋯Br4^v^, symmetry code: (v) *x* − 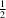, *y* + 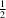, *z*], the average distance of H⋯Br is 3.356 (3) Å and the average value of the O—H⋯Br angle is 161 (4)°. For four N—H⋯Br hydrogen bonds [N1—H1*B*⋯Br4, N2—H2*B*⋯Br1^iii^, N2—H2*A*⋯Br2^iv^, N2—H2*B*⋯Br4, symmetry codes: (iii) −*x* + 1, −*y* + 1, *z* − 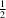; (iv) *x*, −*y* + 1, −*z* + 1], the average H⋯Br distance is 3.468 (3) Å and the average N—H⋯Br angle is 141 (3)°. These values show that hydrogen bonds are quite strong.

## 4. Database survey

A search of the Cambridge Crystallographic Database (updated 20 March 2023; Groom *et al.*, 2016 ▸) using the [RuBr_6_]^2–^ complex ion and the αα′-bis­(di­thio­bis­form­amidinium) cation as the search fragments revealed five closely related compounds, *viz*. bis­[α,α′-di­thio­bis­(form­amidinium)] hexa­bromido­osmium(IV) dibromide trihydrate (CSD refcode XAJVUB; Rudnitskaya *et al.*, 2008 ▸), bis­[disulfane­diyl­bis(amino­methaniminium)] bis­(chloride) hexa­chlorido­osmium(IV) trihydrate (NIPBIA; Rudnitskaya *et al.*, 2019 ▸), bis­[disulfanediylbis(amino­methaniminium)] bis­(bro­mide) hexa­bromido­osmium(IV) trihydrate (NIPBOG; Rudnitskaya *et al.*, 2019 ▸), bis­[(di­amino­methyl­ene)sulfonium] hexa­chlor­ido­osmium (PATCIZ; Rudnitskaya *et al.*, 2017*b* ▸) and bis­[(di­amino­methyl­ene)sulfonium] hexa­bromido­osmium (PATCOF; Rudnitskaya *et al.*, 2017*b* ▸).

XAJVUB, NIPBIA and NIPBOG crystallize in the ortho­rhom­bic *Cmcm* space group with *Z* = 4, while PATCIZ and PATCOF crystallize in the triclinic *P*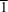 space group with *Z* = 1. In XAJVUB, the [OsBr_6_] ^2–^ anionic complex has an octa­hedral structure. The Os—Br distances fall in the range 2.483–2.490 Å. The α,α′-di­thio­bis­formamidinium cation is a product of the oxidation of thio­carbamide. The S—S and C—S distances are 2.016 and 1.784 Å, respectively. The water mol­ecules, Br^−^ ions, and NH_2_ groups of the cation are linked by hydrogen bonds. In NIPBIA and NIPBOG, the osmium atoms in the [Os*X*_6_] ^2−^ (*X* = Cl or Br) anions adopt slightly distorted octa­hedral coordination. The α,α′-di­thio­bis­formamidinium cations are paired into rings by N—H⋯Cl^−^ hydrogen bonds. The rings are further connected into a 3D framework by hydrogen bonds involving the water mol­ecules and S⋯Cl non-covalent inter­actions. In the crystal structures of PATCIZ and PATCOF, the (NH_2_)_2_CSH^+^ cations and [Os*X*_6_]^2−^ anions are linked into two-tier layers in the (110) plane by the N—H⋯*X*(Cl, Br) and S—H⋯*X*(Cl, Br) hydrogen bonds and S⋯*X*(Cl, Br) non-covalent attractive contacts. It can be seen from the similar geometric parameter values given in Table 3 ▸ that the discussed compounds are comparable with each other.

## 5. Synthesis and crystallization

**Synthesis of [(NH_2_)_2_CSSC(NH_2_)_2_]_2_[RuBr_6_]Br_2_·3H_2_O**

To 0.10 g of K_4_[Ru_2_OCl_10_], 10 mL of concentrated HBr (from Riedel de Haen) were added, and the reaction mixture was heated in a water bath until the K_4_[Ru_2_OCl_10_] dissolved completely. Then a solution of 0.13 g of [S_2_C_2_(NH_2_)_4_]Br_2_·2H_2_O (molar ratio Ru:[S_2_C_2_(NH_2_)_4_]Br_2_ = 1:1.5) was added, and the resulting solution was evaporated in a water bath to 2–3 ml. Dark, large ortho­rhom­bic crystals formed after the solution was cooled. The precipitate was filtered off, and washed with 3 ml of distilled water and 4 ml of ethanol. The mass of the obtained solid was 0.10 g (yield: 67%). Found Ru = 9.6%, for [S_2_C_2_(NH_2_)_4_]_2_[RuBr_6_]Br_2_·3H_2_O: calculated Ru = 9.67%. Sulfur and bromine were determined by microanalysis, and ruthenium was determined by reducing the sample to metallic ruthenium in a stream of H_2_ at 1073 K. Errors between the found and measured values are normal depending on the technique used.

The compound is highly soluble in DMSO, giving blue solutions, while in dilute and concentrated HBr it dissolves over time (red and orange solutions, respectively) and is insoluble in alcohol and acetone.

**EAS** [λ_max_, nm (ɛ, mol^−1^ L cm^−1^)]: in HBr (2 mol L^−1^) – 400 (1800), 460 (1550), 560sh (720); in HBr (9 mol L^−1^) – 375*sh* (2850), 390 (2830), 440*sh* (2050), 455 (2450), 465*sh* (2400), 520*sh* (800); in DMSO – 390*sh* (6700), 480*sh* (2000), 505*sh* (2800), 555 (3930), 619 (4060), 686 (3980), 710sh (3000), 755*sh* (1900).

**FTIR** (ν, cm^−1^): 183, 230 ν (Ru—Br); 422 ρ(SCN_2_), ρ(NH_2_); 497 δ(CN_2_); 577 b(SCN_2_), ν(CS); 815 ν(CS), ν_s_(CN), δ(CN_2_); 1063 ρ(NH_2_), ν_as_(CN); 1119, 1385 ν_s_(CN), ν(CS), ρ(NH_2_); 1621, 1627, 1645 δ(NH_2_), δ(OH_2_), ν_as_(CN); 3050 ν(NH), 3260, 3400 ν_as_(NH), ν(OH).

The reaction with K_4_[Ru_2_OCl_10_] was carried out similarly at a ratio of Ru:[S_2_C_2_(NH_2_)_4_]^2+^ = 1:2, and reactions with ruthenium trichlorides were carried out using ratios of reactants of 1:1.5 and 1:2.

As a result of all experiments, [S_2_C_2_(NH_2_)_4_]_2_[RuBr_6_]Br_2_·3H_2_O precipitates formed, but when the ratio of the initial components was 1:2, the precipitates contained an admixture of [S_2_C_2_(NH_2_)_4_]Br_2_·2H_2_O.

## 6. Refinement

Crystal data, data collection and structure refinement details are summarized in Table 4 ▸. All hydrogen atoms were derived from the Fourier synthesis map and refined isotopically with dependent isotropic thermal parameters with *U*_iso_(H) = 1.2*U*_eq_(N) and *U*_iso_(H) = 1.5*U*_eq_(O).

## Supplementary Material

Crystal structure: contains datablock(s) I. DOI: 10.1107/S2056989024006832/ny2005sup1.cif

Structure factors: contains datablock(s) I. DOI: 10.1107/S2056989024006832/ny2005Isup2.hkl

CCDC reference: 2370115

Additional supporting information:  crystallographic information; 3D view; checkCIF report

## Figures and Tables

**Figure 2:**
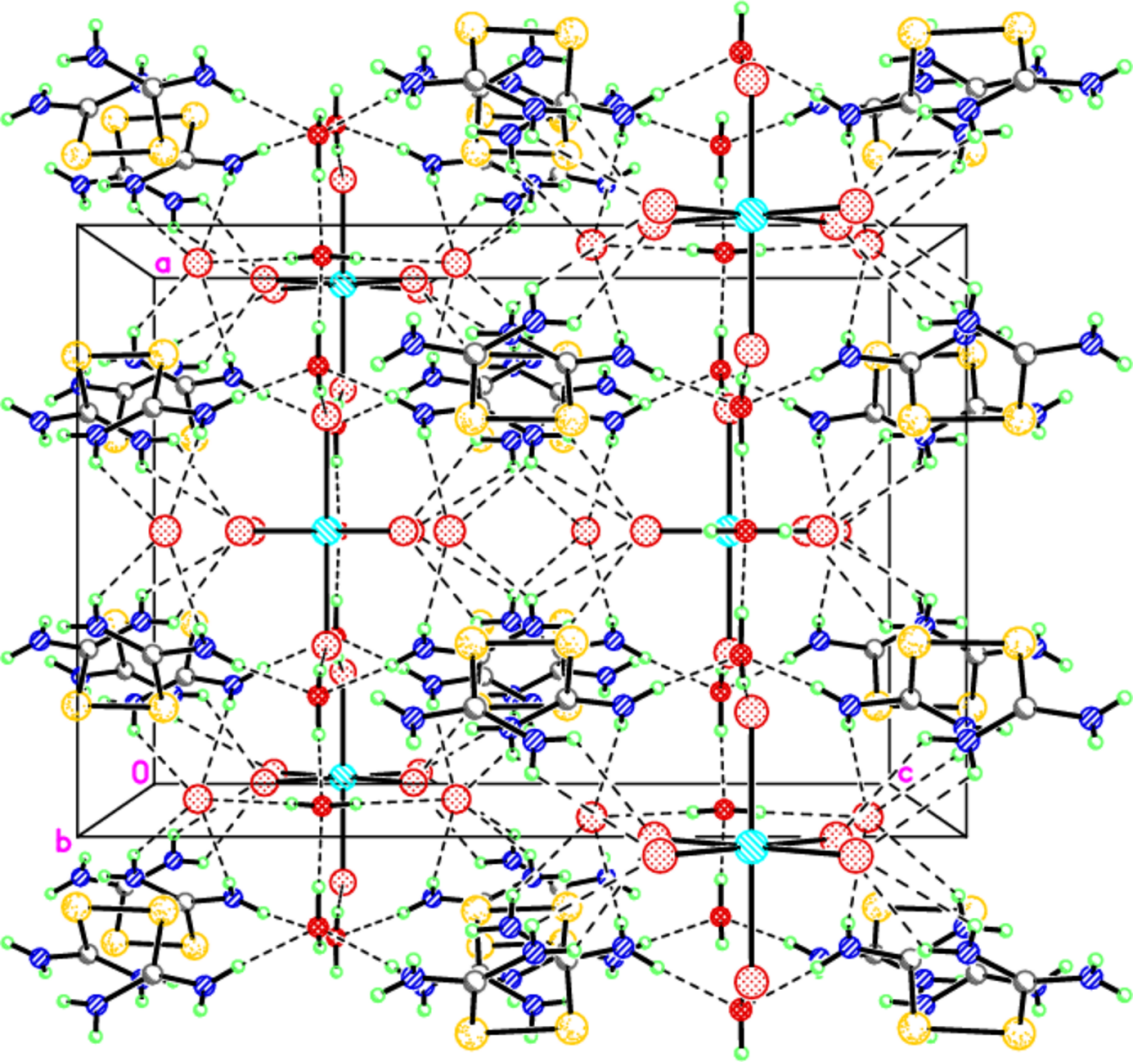
View of the arrangement and inter­actions of the [(NH_2_)_2_CSSC(NH_2_)_2_]^2+^ cations, the RuBr_6_^2–^ and Br^−^ anions, and water mol­ecules in the unit cell.

**Table 1:** Selected bond lengths (Å)

Ru1—Br1	2.4779 (4)	Ru1—Br3	2.4866 (3)
Ru1—Br3^i^	2.4866 (3)	Ru1—Br2	2.4890 (4)

Symmetry code: (i) 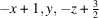.

**Table 2:** Hydrogen-bond geometry (Å, °)

*D*—H⋯*A*	*D*—H	H⋯*A*	*D*⋯*A*	*D*—H⋯*A*
N1—H1*A*⋯O1	0.80 (4)	2.02 (4)	2.814 (3)	167 (3)
O1—H1*C*⋯Br3	0.84 (3)	2.52 (3)	3.293 (3)	154 (4)
O1—H1*D*⋯O2	0.84 (3)	2.00 (4)	2.794 (4)	157 (5)
N1—H1*B*⋯Br4	0.77 (3)	2.60 (4)	3.327 (2)	157 (3)
N2—H2*B*⋯Br1^ii^	0.81 (3)	2.96 (4)	3.496 (2)	125 (3)
N2—H2*A*⋯Br2^iii^	0.88 (4)	2.79 (4)	3.456 (2)	134 (3)
N2—H2*B*⋯Br4	0.81 (3)	2.87 (4)	3.592 (3)	149 (3)
O2—H2⋯Br4^iv^	0.95 (5)	2.48 (5)	3.419 (2)	167 (4)

Symmetry codes: (ii) 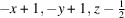; (iii) 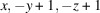; (iv) 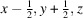.

**Table 3:** Selected values of bond distances (Å) and angles (°) in various salts and complexes

Compound	S—S	C—S	C—S—S	C—S—S—C	Reference
Title compound	2.0282 (12)	1.783 (2)	102.90 (9)	−95.15 (11)	This study
XAJVUB	2.024 (2)	1.778 (5)	103.2 (2)	96.3	(Rudnitskaya *et al.*, 2008 ▸)
NIPBIA	2.036 (2)	1.789 (5)	102.43 (17)	−96.4 (4)	(Rudnitskaya *et al.*, 2019 ▸)
NIPBOG	2.039 (2)	1.796 (5)	102.62 (15)	−97.9 (3)	(Rudnitskaya *et al.*, 2019 ▸)
PATCIZ	–	1.739 (10)	–	–	(Rudnitskaya *et al.*, 2017*b* ▸)
PATCOF	–	1.751 (9)	–	–	(Rudnitskaya *et al.*, 2017*b* ▸)

**Table 4:** Experimental details

Crystal data	
Chemical formula	(C_2_H_8_N_4_S_2_)_2_[RuBr_6_]Br_2_·3H_2_O
*M*_r_	1098.88
Crystal system, space group	Orthorhombic, *Cmcm*
Temperature (K)	150
*a*, *b*, *c* (Å)	11.6462 (3), 13.9943 (4), 16.9225 (5)
*V* (Å^3^)	2758.04 (13)
*Z*	4
Radiation type	Mo *K*α
μ (mm^−1^)	12.49
Crystal size (mm)	0.30 × 0.20 × 0.02

Data collection	
Diffractometer	Bruker D8 Venture
Absorption correction	Multi-scan (*SADABS*; Krause *et al.*, 2015 ▸)
*T*_min_, *T*_max_	0.043, 0.100
No. of measured, independent and observed [*I* > 2σ(*I*)] reflections	22642, 2269, 2057
*R*_int_	0.043
(sin θ/λ)_max_ (Å^−1^)	0.715

Refinement	
*R*[*F*^2^ > 2σ(*F*^2^)], *wR*(*F*^2^), *S*	0.023, 0.057, 1.07
No. of reflections	2269
No. of parameters	93
No. of restraints	1
H-atom treatment	Only H-atom coordinates refined
Δρ_max_, Δρ_min_ (e Å^−3^)	1.06, −0.59

Computer programs: *APEX3* and *SAINT* (Bruker, 2016 ▸), *SHELXT2014/5* (Sheldrick, 2015*a* ▸), *SHELXL2019/3* (Sheldrick, 2015*b* ▸), *ORTEP-3 for Windows* (Farrugia, 2012 ▸) and *PLATON* (Spek, 2020 ▸).

## supplementary crystallographic information

### Bis[dithiobis(formamidinium)] hexabromidoruthenium dibromide trihydrate Crystal data

**Table:**

(C_2_H_8_N_4_S_2_)_2_[RuBr_6_]Br_2_·3H_2_O	*D*_x_ = 2.646 Mg m^−^^3^
*M**_r_* = 1098.88	Mo *K*α radiation, λ = 0.71073 Å
Orthorhombic, *C**m**c**m*	Cell parameters from 1291 reflections
*a* = 11.6462 (3) Å	θ = 2.3–26.6°
*b* = 13.9943 (4) Å	µ = 12.49 mm^−^^1^
*c* = 16.9225 (5) Å	*T* = 150 K
*V* = 2758.04 (13) Å^3^	Plate, black
*Z* = 4	0.30 × 0.20 × 0.02 mm
*F*(000) = 2056	

### Bis[dithiobis(formamidinium)] hexabromidoruthenium dibromide trihydrate Data collection

**Table:**

Bruker D8 Venture diffractometer	2057 reflections with *I* > 2σ(*I*)
Radiation source: microsource	*R*_int_ = 0.043
φ and ω scans	θ_max_ = 30.5°, θ_min_ = 2.3°
Absorption correction: multi-scan (SADABS; Krause *et al.*, 2015)	*h* = −16→16
*T*_min_ = 0.043, *T*_max_ = 0.100	*k* = −19→20
22642 measured reflections	*l* = −24→24
2269 independent reflections	

### Bis[dithiobis(formamidinium)] hexabromidoruthenium dibromide trihydrate Refinement

**Table:**

Refinement on *F*^2^	Primary atom site location: difference Fourier map
Least-squares matrix: full	Secondary atom site location: difference Fourier map
*R*[*F*^2^ > 2σ(*F*^2^)] = 0.023	Hydrogen site location: difference Fourier map
*wR*(*F*^2^) = 0.057	Only H-atom coordinates refined
*S* = 1.07	*w* = 1/[σ^2^(*F*_o_^2^) + (0.0237*P*)^2^ + 6.898*P*] where *P* = (*F*_o_^2^ + 2*F*_c_^2^)/3
2269 reflections	(Δ/σ)_max_ = 0.001
93 parameters	Δρ_max_ = 1.06 e Å^−^^3^
1 restraint	Δρ_min_ = −0.59 e Å^−^^3^

### Bis[dithiobis(formamidinium)] hexabromidoruthenium dibromide trihydrate Special details

**Table:**

Geometry. All esds (except the esd in the dihedral angle between two l.s. planes) are estimated using the full covariance matrix. The cell esds are taken into account individually in the estimation of esds in distances, angles and torsion angles; correlations between esds in cell parameters are only used when they are defined by crystal symmetry. An approximate (isotropic) treatment of cell esds is used for estimating esds involving l.s. planes.

### Bis[dithiobis(formamidinium)] hexabromidoruthenium dibromide trihydrate Fractional atomic coordinates and isotropic or equivalent isotropic displacement parameters (Å^2^)

**Table:**

	*x*	*y*	*z*	*U*_iso_*/*U*_eq_	
Ru1	0.500000	0.65423 (2)	0.750000	0.01754 (8)	
Br1	0.500000	0.77930 (2)	0.85365 (2)	0.02178 (8)	
Br2	0.500000	0.52583 (2)	0.64822 (2)	0.02559 (8)	
Br3	0.28649 (3)	0.65551 (2)	0.750000	0.02565 (9)	
Br4	0.500000	0.17169 (2)	0.58416 (2)	0.02717 (9)	
S1	0.18284 (5)	0.47494 (4)	0.55623 (4)	0.02521 (13)	
N1	0.2957 (2)	0.33273 (17)	0.61812 (15)	0.0280 (5)	
H1A	0.264 (3)	0.353 (2)	0.657 (2)	0.034*	
H1B	0.330 (3)	0.285 (2)	0.616 (2)	0.034*	
N2	0.3321 (2)	0.35163 (17)	0.48633 (16)	0.0329 (5)	
H2A	0.331 (3)	0.386 (3)	0.443 (2)	0.039*	
H2B	0.380 (3)	0.310 (2)	0.489 (2)	0.039*	
C1	0.2816 (2)	0.37771 (17)	0.55165 (16)	0.0241 (5)	
O1	0.2061 (2)	0.4299 (2)	0.750000	0.0305 (6)	
H1C	0.249 (4)	0.478 (3)	0.750000	0.046*	
H1D	0.136 (3)	0.445 (4)	0.750000	0.046*	
O2	0.000000	0.5321 (3)	0.750000	0.0360 (9)	
H2	0.000000	0.579 (4)	0.709 (3)	0.054*	

### Bis[dithiobis(formamidinium)] hexabromidoruthenium dibromide trihydrate Atomic displacement parameters (Å^2^)

**Table:**

	*U*^11^	*U*^22^	*U*^33^	*U*^12^	*U*^13^	*U*^23^
Ru1	0.01735 (16)	0.01668 (16)	0.01860 (17)	0.000	0.000	0.000
Br1	0.02324 (15)	0.02066 (15)	0.02144 (16)	0.000	0.000	−0.00283 (11)
Br2	0.03078 (18)	0.02150 (16)	0.02447 (17)	0.000	0.000	−0.00468 (12)
Br3	0.01777 (15)	0.02213 (16)	0.0371 (2)	−0.00096 (11)	0.000	0.000
Br4	0.02496 (16)	0.02304 (16)	0.0335 (2)	0.000	0.000	0.00104 (13)
S1	0.0280 (3)	0.0238 (3)	0.0238 (3)	0.0043 (2)	0.0033 (2)	0.0016 (2)
N1	0.0287 (11)	0.0256 (11)	0.0298 (12)	0.0040 (8)	0.0033 (9)	0.0043 (9)
N2	0.0370 (13)	0.0272 (11)	0.0345 (13)	0.0063 (10)	0.0128 (11)	0.0034 (10)
C1	0.0228 (11)	0.0199 (10)	0.0295 (13)	−0.0019 (9)	0.0023 (9)	0.0015 (9)
O1	0.0329 (14)	0.0254 (13)	0.0333 (15)	0.0003 (11)	0.000	0.000
O2	0.042 (2)	0.032 (2)	0.035 (2)	0.000	0.000	0.000

### Bis[dithiobis(formamidinium)] hexabromidoruthenium dibromide trihydrate Geometric parameters (Å, º)

**Table:**

Ru1—Br1	2.4779 (4)	N1—H1A	0.80 (4)
Ru1—Br1^i^	2.4779 (4)	N1—H1B	0.77 (3)
Ru1—Br3^ii^	2.4866 (3)	N2—C1	1.305 (3)
Ru1—Br3	2.4866 (3)	N2—H2A	0.88 (4)
Ru1—Br2^i^	2.4890 (4)	N2—H2B	0.81 (3)
Ru1—Br2	2.4890 (4)	O1—H1C	0.84 (3)
S1—C1	1.783 (2)	O1—H1D	0.84 (3)
S1—S1^iii^	2.0282 (12)	O2—H2	0.95 (5)
N1—C1	1.299 (3)		
			
Br1—Ru1—Br1^i^	90.122 (19)	Br3—Ru1—Br2	90.298 (8)
Br1—Ru1—Br3^ii^	89.708 (8)	Br2^i^—Ru1—Br2	87.58 (2)
Br1^i^—Ru1—Br3^ii^	89.708 (8)	C1—S1—S1^iii^	102.90 (9)
Br1—Ru1—Br3	89.708 (8)	C1—N1—H1A	119 (2)
Br1^i^—Ru1—Br3	89.708 (8)	C1—N1—H1B	117 (3)
Br3^ii^—Ru1—Br3	179.17 (2)	H1A—N1—H1B	124 (4)
Br1—Ru1—Br2^i^	91.151 (12)	C1—N2—H2A	123 (2)
Br1^i^—Ru1—Br2^i^	178.727 (16)	C1—N2—H2B	117 (3)
Br3^ii^—Ru1—Br2^i^	90.298 (8)	H2A—N2—H2B	117 (3)
Br3—Ru1—Br2^i^	90.298 (8)	N1—C1—N2	122.7 (2)
Br1—Ru1—Br2	178.729 (16)	N1—C1—S1	114.4 (2)
Br1^i^—Ru1—Br2	91.150 (12)	N2—C1—S1	122.8 (2)
Br3^ii^—Ru1—Br2	90.298 (8)	H1C—O1—H1D	112 (5)
			
S1^iii^—S1—C1—N1	−179.53 (18)	S1^iii^—S1—C1—N2	−2.0 (2)

Symmetry codes: (i) *x*, *y*, −*z*+3/2; (ii) −*x*+1, *y*, −*z*+3/2; (iii) *x*, −*y*+1, −*z*+1.

### Bis[dithiobis(formamidinium)] hexabromidoruthenium dibromide trihydrate Hydrogen-bond geometry (Å, º)

**Table:**

*D*—H···*A*	*D*—H	H···*A*	*D*···*A*	*D*—H···*A*
N1—H1*A*···O1	0.80 (4)	2.02 (4)	2.814 (3)	167 (3)
O1—H1*C*···Br3	0.84 (3)	2.52 (3)	3.293 (3)	154 (4)
O1—H1*D*···O2	0.84 (3)	2.00 (4)	2.794 (4)	157 (5)
N1—H1*B*···Br4	0.77 (3)	2.60 (4)	3.327 (2)	157 (3)
N2—H2*B*···Br1^iv^	0.81 (3)	2.96 (4)	3.496 (2)	125 (3)
N2—H2*A*···Br2^iii^	0.88 (4)	2.79 (4)	3.456 (2)	134 (3)
N2—H2*B*···Br4	0.81 (3)	2.87 (4)	3.592 (3)	149 (3)
O2—H2···Br4^v^	0.95 (5)	2.48 (5)	3.419 (2)	167 (4)

Symmetry codes: (iii) *x*, −*y*+1, −*z*+1; (iv) −*x*+1, −*y*+1, *z*−1/2; (v) *x*−1/2, *y*+1/2, *z*.
